# Dramatic Declines of Montane Frogs in a Central African Biodiversity Hotspot

**DOI:** 10.1371/journal.pone.0155129

**Published:** 2016-05-05

**Authors:** Mareike Hirschfeld, David C. Blackburn, Thomas M. Doherty-Bone, LeGrand Nono Gonwouo, Sonia Ghose, Mark-Oliver Rödel

**Affiliations:** 1 Museum für Naturkunde Berlin, Leibniz Institute for Evolution and Biodiversity Science, Invalidenstraße 43, 10115 Berlin, Germany; 2 Department of Herpetology, California Academy of Sciences, San Francisco, California 94118, United States of America; 3 Conservation Research & Action for Amphibians of Cameroon, Royal Zoological Society of Scotland, Edinburgh Zoo, Edinburgh, United Kingdom; 4 Cameroon Herpetology-Conservation Biology Foundation (CAMHERP-CBF), PO Box 8218, Yaoundé, Cameroon; Vanderbilt University School of Medicine, UNITED STATES

## Abstract

Amphibian populations are vanishing worldwide. Declines and extinctions of many populations have been attributed to chytridiomycosis, a disease induced by the pathogenic fungus *Batrachochytrium dendrobatidis* (*Bd*). In Africa, however, changes in amphibian assemblages were typically attributed to habitat change. We conducted a retrospective study utilizing field surveys from 2004–2012 of the anuran faunas on two mountains in western Cameroon, a hotspot of African amphibian diversity. The number of species detected was negatively influenced by year, habitat degradation, and elevation, and we detected a decline of certain species. Because another study in this region revealed an emergence of *Bd* in 2008, we screened additional recent field-collected samples and also pre-decline preserved museum specimens for the presence of *Bd* supporting emergence before 2008. When comparing the years before and after *Bd* detection, we found significantly diminished frog species richness and abundance on both mountains after *Bd* emergence. Our analyses suggest that this may be the first disease-driven community-level decline in anuran biodiversity in Central Africa. The disappearance of several species known to tolerate habitat degradation, and a trend of stronger declines at higher elevations, are consistent with *Bd*-induced declines in other regions. Not all species decreased; populations of some species remained constant, and others increased after the emergence of *Bd*. This variation might be explained by species-specific differences in infection probability. Increased habitat protection and *Bd*-mitigation strategies are needed for sustaining diverse amphibian communities such as those on Mt. Manengouba, which contains nearly half of Cameroon’s frog diversity.

## Introduction

Causes of current global amphibian declines are manifold, including habitat loss and fragmentation, environmental pollution, climate change, and emerging infectious diseases like chytridiomycosis caused by chytrid fungi *Batrachochytrium dendrobatidis and B*. *salamandrivorans* [1–5]. Severe population declines of amphibians are generally the consequence of an interaction of several of the aforementioned factors. The outbreak of chytridiomycosis is often linked to weather conditions [6] and season [7]. The most studied pathogenic fungus in amphibians, *B*. *dendrobatitidis* (*Bd*), is distributed worldwide [8], and amphibians have been particularly impacted in Central America and Australia [9–11]. Although amphibians across much of continental Africa (summarized in reference [12]) and Madagascar [13,14] have tested positive for *Bd*, no large-scale declines caused by the disease have been reported. In Africa, changes in local amphibian species composition, richness, and abundance are usually attributed to habitat change [15–20]. Only a few declines of a single species in Tanzania [21,22] and Lesotho [23] were assumed to be related to *Bd*.

Cameroon is among the richest countries for amphibian diversity, and its volcanic line is one of Africa’s most important biodiversity hotspots [24]. Field observations over the past decade indicate that several montane frog species have become difficult or even impossible to find, and recent studies suggest an emergence of *Bd* in the mountains of Cameroon around 2008 [25,26]. Here, we report a decline in montane frog species. We test if this decline can be robustly explained by habitat disturbance or the emergence of *Bd*. We utilize extensive data from amphibian surveys collected from 2004–2012 on two mountains with rich endemic anuran faunas. We test whether the number of species detected is related to environmental factors or study year and investigate whether the frequencies of particular genera and species differ between surveys before and after the first detection of *Bd* on these mountains.

## Materials and Methods

### Ethics statement

No Institutional Animal Care and Use Committee (IACUC) or ethics committee approved this study as this was not required by Cameroonian law. However, all work complied with the guidelines for the use of live amphibians and reptiles in field research compiled by the American Society of Ichthyologists and Herpetologists (ASIH), The Herpetologists' League (HL) and the Society for the Study of Amphibians and Reptiles (SSAR).

The amphibian data presented here are compiled from several studies to examine the taxonomy of species present in Cameroon. Species collected for related studies from our study sites and nearby sites and later tested for *Bd* are included and listed in S1 Table.

For the amphibian survey, individuals were identified to species level in the field and immediately released at the same site. In cases of taxonomic uncertainty, frogs and tadpoles were collected as vouchers for later identification. In those instances, individuals were anesthetised and euthanized using a water bath with either MS-222 (tricaine methanesulfonate) or chlorobutanol, both recommended for painlessly killing amphibians. For additional *Bd* screenings in the field, we used epithelial swabbing. Cotton swabs were utilised to brush the skin of living amphibians.

None of the species involved in our study are protected by any national or international law. The land where our study took place is publicly owned, and access is controlled by the community. We only worked in an area after permission by the person in authority (e.g. village chiefs, community elders). Study sites were either located in non-protected areas or the access to protected sites (Kilum-Ijim Plantlife Sanctuary, Mt. Oku) was explicitly allowed by permit.

Research permits covered all areas visited and the entire study protocol, including euthanasia, collection, and the export of species. They were issued by the Cameroonian Ministry of Scientific Research and Innovation (MINRESI) and the Ministry of Forestry and Wildlife (MINFOF).

### Study area

**Fig 1:**
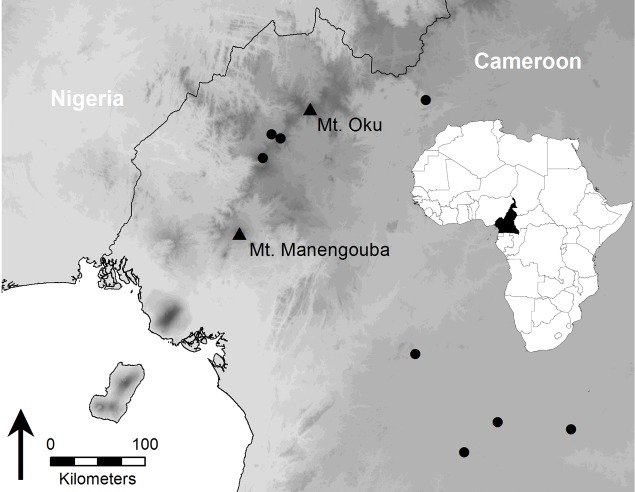
Map of the study region. Locations of the study sites, Mt. Manengouba and Mt. Oku, are marked with black triangles, additional localities surveyed for *Bd* are represented by black dots; map based on SRTM elevation model (light = low, dark = high elevations) [27].

We focused on anuran assemblages from two major peaks of the Cameroon Volcanic Line (Fig 1): Mount Manengouba (2411 meters above sea level-MASL) in southwestern Cameroon, and Mount Oku (3011 MASL) in the North-West Region. At lower elevations, both mountains are characterized by habitats strongly impacted by human activity, e.g. villages, fields, and plantations. At higher elevations, variously sized remnants of montane cloud forests and grasslands (the latter mostly used for grazing livestock) dominate the landscape. Our surveys covered a large elevational and geographical range on both Mt. Manengouba (620–2244 m asl; 4°49'–5°05'N; 9°39'–9°53'E) and Mt. Oku (1569–3011 m asl; 6°09'–6°17'N; 10°19'–10°31'E). Sampling focused on riparian habitats, but also included forested areas lacking standing waters or streams, as well as near crater lakes on both mountains.

### Amphibian surveys

Our study is based on combining data from surveys conducted by researchers that were working independently with differing goals and methods, and thus some levels of granularity in the data are unavoidable. From 2004 to 2012, we repeatedly surveyed both mountains during wet (May–September) and dry (October–April) seasons. We determined the presence of adult anuran species using visual and acoustic encounter surveys during day and night searches. Visually detected frogs were caught by hand. LNG, MH, DCB, and MOR collected data on Mt. Manengouba, and TMD-B, DCB, and LNG on Mt. Oku. The search methods comprised plot searches, transect walks, and incidental searches. In addition, qualitative searches for tadpoles using dip nets were applied in both regions. For our analyses we defined each distinct search per site, independent of method, as one “search event”. On Mt. Manengouba, a total of 366 search events were conducted between 2004 and 2012 (2004: 5, 2005: 25, 2006: 66, 2007: 31, 2008: 70, 2010: 71, 2011: 63, 2012: 35). On Mt. Oku we conducted 193 search events during the same period (2004: 2, 2006: 97, 2008: 18, 2009: 21, 2010: 3, 2012: 52). We recorded date, GPS coordinates, elevation, number of surveyors, search time, and the species detected at each location. At each site we assigned the habitat to a general category (primary forest, secondary forest, strongly disturbed and open habitat, village, montane grassland) and noted the availability of nearby ponds and streams. Habitat categories as well as the presence of a pond and stream were binary coded (true or false) for each search event. Collected vouchers (see above) of frogs and tadpoles are stored at the Museum für Naturkunde Berlin (ZMB), Natural History Museum London (BMNH), the Museum of Comparative Zoology (MCZ), and in Yaoundé (foundation for a future national reference collection).

### *Bd* screenings

To supplement recently published surveys of *Bd* in Cameroon [25,26,28,29], we assessed the occurrence of *Bd* in Cameroon using both preserved museum specimens collected from 1908 to 2006 and additional field collected samples from 2011 and 2013. Field samples were collected on Mt. Manengouba and Mt. Oku, as well as from many other sites in the country including highland areas and lower elevation localities (Fig 1). Historical samples were taken from museum specimens collected from the same or nearby sites. All museum-preserved specimens were housed at the California Academy of Sciences (CAS) or at the Museum of Comparative Zoology at Harvard University (MCZ). Importantly, many records in Doherty-Bone et al. [26] and museum specimens collected in 2004 and 2006 by DCB correspond to the field records used in this study on declines.

We used standard skin swabbing techniques to test for the presence of *Bd* [30–32]. Using a MW113 dry swab (Medical Wire and Equipment Company), all specimens were stroked 30 times. This included ten strokes of each side of the ventrum, and five strokes on each of the hind feet, focusing on sampling between the toes. Field swabs were then placed in microcentrifuge tubes (1.5 ml) containing 70% ethanol. Gloves were used during collection of swabs from museum specimens, and changed between handling every specimen. In addition, to decrease the chance of cross contamination between museum specimens housed in shared jars, each specimen was rinsed in 70% ethanol prior to swabbing. Museum specimen swab samples were stored in microcentrifuge tubes without alcohol. Once collected, we attempted to keep swabs as cold as possible in the field and the lab (ideally 4°C) until DNA extraction and quantitative PCR [33]. Prior to DNA extraction, we dried swab samples by spinning in a centrifuge under vacuum (SpeedVac™). We extracted DNA using the Prepman™ Ultra Sample Preparation Reagent (Life Technologies), and conducted real-time quantitative PCR according to standard methods [30,31]. These methods for DNA extraction and qPCR have been validated both in live specimens and formalin-fixed museum specimens stored in 70% ethanol [32,34]. Genomic equivalent (GE) results were multiplied by the dilution factor of 80 to estimate the number of zoospores in a swab, or zoospore equivalent (ZE).

### Data analysis

Sampling effort was measured in person-hours and calculated as the number of surveyors multiplied by search time. We calculated the sampling success expressed as the number of species recorded per person-hour for each search event. As survey time was not available for all incidental searches, the sample size of values related to time (sampling effort, sampling success) was smaller than the overall number of search events.

To investigate whether sampling success was influenced by one or a combination of the selected factors (habitat category, availability of ponds or streams, study year, elevation) and their interactions (study year*elevation to test for differences along the elevation with time), a generalized mixed-effect model was calculated (function glmer, R package 'lme4' [35]). Numeric variables (elevation, study year) were scaled from 0 to 1. We corrected for possible differences among both mountains by adding the study area (Mt. Manengouba or Mt. Oku) as a random factor to the model. The model was reduced stepwise backwards based on the Akaike information criterion (AIC). To test for an influence of the emergence of *Bd*, the data were grouped by whether the search events occurred before or after the first detection of *Bd* on each mountain. To assess species-level responses, we calculated the frequency of occurrence for a species as the number of search events detecting the species, divided by the total number of search events for each year and the period before and after *Bd* occurrence, respectively. A similar approach was applied for each genus.

Our data (Shapiro-Wilk test: species per sampling event: W = 0.90, p < 0.0001; sampling success: W = 0.82, p < 0.0001; R package ‘stats’) and their residuals (visually tested using QQ plots, R package ‘stats’) were not normally distributed. We thus applied Pearson’s correlation coefficient to test for dependencies, the Wilcoxon rank-sum test for pairwise comparisons before and after *Bd* detection on each mountain and a generalized mixed-effect model for negative binomial distribution (differences from negative binomial: species per sampling event: χ² = 7.58, df = 10, p = 0.06; sampling success: χ² = 4.09, df = 4, p = 0.39; function goodfit, R package ‘vcd’). All statistical analyses were conducted in R 3.1.1 (R Development Core Team).

## Results

### Species richness and sampling success

In total, 89 frog species representing 21 genera were detected on Mt. Manengouba. These comprise all species that have been documented historically [36–38] as well as species recently described from this mountain [39–41]. On average, 3.55 ± 2.32 (mean ± SD, range: 0–15, n = 366) species were detected per search event. Survey effort ranged from 0.01 to 20 person-hours (3.56 ± 2.56, n = 291) and sampling success ranged from 0 to 6.5 (1.21 ± 0.98, n = 291). On Mt. Oku, 28 frog species representing 14 genera with a mean of 1.10 ± 0.99 species per search event (range: 0–6, n = 193) were detected. As for Mt. Manengouba, our surveys documented all previously known species for Mt. Oku as well as new species [41]. The person-hours spent during a search event varied from 0.25 to 19.5 (3.81 ± 3.00, n = 84). The sampling success ranged from 0 to 4 (0.50 ± 0.73, n = 84).

We focused subsequent analysis on sampling success only as this measure takes sampling effort into account and was moreover highly correlated with the number of species detected (cor = 0.66, df = 373, p < 0.0001, n = 375). Sampling success decreased with elevation, study year and the availability of ponds; it decreased with habitat categories ‘village’, ‘secondary forest’, ‘primary forest’ as well as ‘mountainous grassland’ (final generalized mixed-effect model, ΔAIC = 4.8, Table 1).

**Table 1:** Effects of habitat and study year on species sampling success.

		Fixed effects		
	Estimate	Standard error	T statistics	P
(Intercept)	1.6407	0.1668	9.834	< 0.0001
Elevation	-1.4995	0.2810	-5.337	< 0.0001
Year	-1.0631	0.2043	-5.204	< 0.0001
Pond	0.4653	0.1801	2.583	< 0.01
Village	-1.0927	0.2681	-4.075	< 0.0001
Secondary forest	-0.4467	0.1387	-3.221	< 0.01
Primary forest	-0.7706	0.2234	-3.450	< 0.001
Mountainous grassland	-0.2234	0.1392	1.605	> 0.05

Independent variables included elevation, habitat categories (primary forest, secondary forest, strongly disturbed and open habitat, village, and mountainous grassland), study year, and the availability of ponds and streams. The study site was added as a random factor and the best-fit model is presented; a mixed-effect model analysis was conducted using function glmer.nb in package ‘lme4’ in R 3.1.1 [35], models were reduced stepwise backwards using AIC (maximum likelihood), ΔAIC = 4.8 (full model = 857.1, reduced model = 852.3).

### *Bd* screenings

We tested 531 preserved specimens and 1271 additional recent field-collected samples for *Bd* (S2 Table and S3 Table). Combined with published *Bd* screenings (see Materials and Methods), these data provide a chronology of the occurrence of *Bd* across Cameroon. *Bd* was first detected in the lowlands at Batouri in 1934 and again in the lowlands near Sangmelima in 1960, though both times at low infection intensities. There are no *Bd*-positive records in the highlands of Cameroon until 2008 (S3 Table), although sample sizes were frequently below the threshold required to robustly infer prevalence below 1% (*n* = 298) or even 5% (*n* = 59) [42]. In total, 309 frogs tested positive for *Bd* with genomic equivalent scores ranging from 0.0001 to 517.46 (7.11 ± 38.71, n = 309, S2 Table and S3 Table for details). *Bd* was first detected in samples from Mt. Manengouba in 2011 and from Mt. Oku in 2008, supporting Doherty-Bone et al. [25].

### *Bd* detection and sampling success

**Fig 2:**
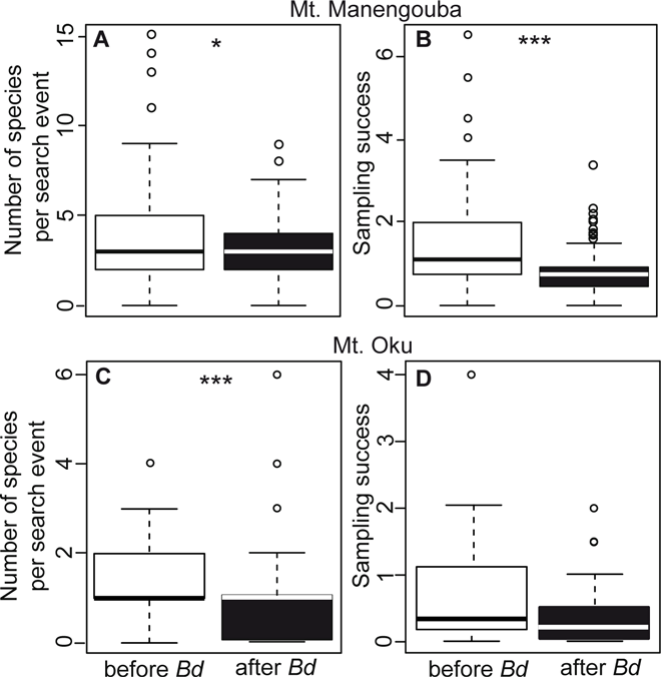
Differences in detected anuran species between search events conducted before and after *Bd*-positive records in these study regions. (A) Number of species per sampling event at Mt. Manengouba. (B) Sampling success (number of species recorded per person-hour for each search event at Mt. Manengouba. (C) Number of species per sampling event at Mt. Oku. (D) Sampling success at Mt. Oku. Boxplots depicting events before the detection of *Bd* in the region are shown as colored white, and those representing events after detection of *Bd* are shown as colored black. See Table 2 for statistical results and sample sizes

To test for the influence of *Bd* on the low number of species detected in recent years, we combined search events based on whether they were conducted before or after the first detection of *Bd* on each mountain. On Mt. Manengouba, we observed 86 species before and only 40 after the first detection of *Bd*. The sampling success and the number of species observed per search event decreased significantly after the detection (Table 2; Fig 2A and 2B). Similarly on Mt. Oku we found fewer species (18 versus 24) and significantly fewer species per sampling event (Table 2, Fig 2C) after the first positive *Bd* record compared to before. Sampling success was also higher before *Bd* detection, although not significantly (Table 2, Fig 2D).

**Table 2:** Differences in sampling success and species occurrence before and after the first positive *Bd* record on Mt. Manengouba in 2011 and Mt. Oku in 2008.

	Before *Bd*		After *Bd*		Wilcoxon rank sum test	
	Mean ± SD	n	Mean ± SD	N	*W*	P-value
Sampling Success^a^						
Mt. Manengouba	1.44 ± 1.07	194	0.76±0.56	97	13946.5	< 0.0001
Mt. Oku	0.70 ± 0.97	36	0.35±0.44	48	1058	> 0.05
Number of Species per Search Event						
Mt. Manengouba	3.74 ± 2.41	268	3.04±1.97	98	15258.5	< 0.05
Mt. Oku	1.31 ± 086	99	0.88±1.09	94	6128	< 0.0001

Given are the mean, standard deviation (mean ± SD), and sample size (n, search events) before and after *Bd*, and results of the Wilcoxon rank sum-test.

^a^ number of species recorded per person-hour for each search event.

### Detection trends of specific species and genera

**Fig 3:**
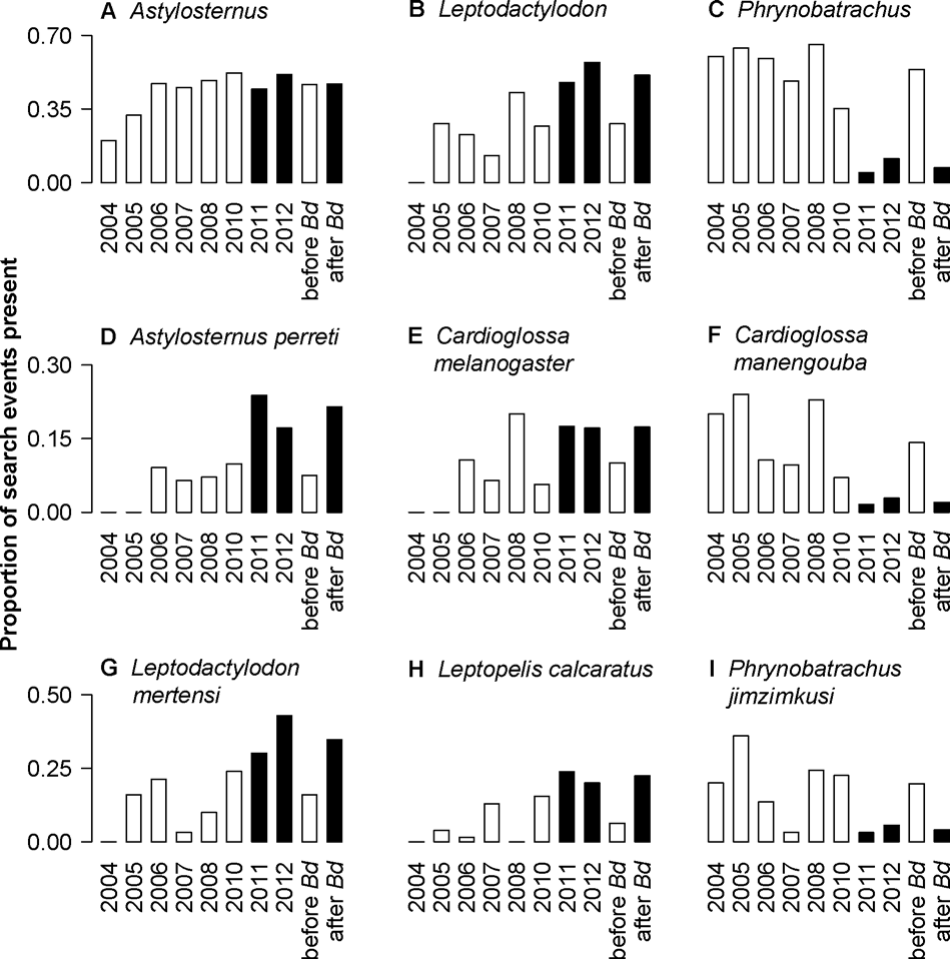
Frequency of detection of selected frog genera and species during all study years on Mt. Manengouba. Shown is the proportion of search events in which a genus (A-C) or a species (D-G) was present in a given year. The latter two bars show search events in which a genus or species was detected in all search events before and after the first recorded detection of *Bd* in the region (compare S3 Table). Bars showing events before the detection of *Bd* are colored white and those showing events after first detection of *Bd* are colored black. The number of search events was as follows: 2004 = 5; 2005 = 25; 2006 = 66; 2007 = 31; 2008 = 70; 2010 = 71; 2011 = 63; 2–12 = 35. Search events prior to detection of *Bd* = 366 and after detection of *Bd* = 98. For additional genus and species information, see S2 Fig.

Although overall fewer species were detected in recent years, not all species were affected similarly. Some species were observed less frequently, but others showed little difference or even increased frequency of observations (see Fig 3 and S2 Fig). On Mt. Manengouba, puddle frogs (*Phrynobatrachus*) were common until 2010, but observations decreased dramatically in the years 2011 and 2012 (Fig 3C). In contrast night frogs (*Astylosternus*) were found in approximately half of the search events in all study years on Mt. Manengouba (Fig 3A). Egg frogs (*Leptodactylodon*) were found more frequently after the emergence of *Bd* (Fig 3B). Although the frequency of single species observed varied among study years, some species appeared to have decreased (e.g. *Cardioglossa manengouba*, *Phrynobatrachus jimzimkusi*) whereas others appeared to have increased (e.g. *Astylosternus perreti*, *Leptodactylodon mertensi*, *Leptopelis calcaratus*) over time (Fig 3D–3F, Figures A and B in S2 Fig). On Mt. Oku, species were detected less frequently. If only considering those species recorded at least 10 times across all sampling events, two genera, squeakers (*Arthroleptis*) and *Phrynobatrachus*, exhibited the largest apparent declines when comparing search events before and after *Bd*-emergence, whereas *Astylosternus* slightly increased. When considering specific species on Mt. Oku, one night frog (*Astylosternus rheophilus*) and two puddle frogs (*Phrynobatrachus steindachneri-jimzimkusi* complex, *P*. *werneri*) appeared to have declined, whereas *Astylosternus ranoides* appeared to have increased considerably (Figure D in S2 Fig). Though caecilians were not the focus of field work, *Crotaphatrema lamottei* has been observed (2012 and 2013) on Mt. Oku following declines of anurans, which suggests that the population of this rare species has not changed [43] in relation to previously common anuran species that have disappeared. Though the amphibian fauna differs between the study sites, frogs inhabiting both mountains likewise decreased (e.g. *P*. *steindachneri-jimzimkusi* complex, *P*. *werneri*).

## Discussion

Our study reveals the first community-level declines in numbers of observable African amphibians associated with an increase of *Bd* prevalence rather than dramatic changes in land use or loss of habitat. Although the decline of a few other amphibians in Africa has been associated with this pathogen, *Bd* does not seem to have negatively affected other species in these faunas. The abrupt decrease in species occurrence and richness described here is comparable with *Bd*-induced amphibian declines on other continents. The magnitude of the apparent decline, however, differs by being less at our Central African study sites. For example, we did not detect large numbers of dead frogs as reported from the Neotropics [10] and Australia [9,10,44]. However, villagers around Mt. Manengouba have reported observing dead frogs on several occasions along different local rivers (unpublished data), but the cause of these mortality events remain unknown. Natural habitats on Mt. Manengouba and Mt. Oku are threatened, mainly due to loss (slash and burn for farming) and degradation (wood-cutting, encroachment by agriculture, livestock grazing). In our study, the strength of habitat disturbance had no clear effect on sampling success. Sampling success likewise increased with pristine (‘primary forest’) and strongly altered (‘village’) habitat categories. Following this, and supported by the decline of species previously common in strongly degraded habitats and not dependent on pristine forests (e.g. *Phrynobatrachus jimzimkusi*), anthropogenic habitat conversion cannot explain the strong declines in the past few years.

The application of pesticides can be lethal to amphibians [45,46] and is common on both mountains studied here (personal observations). However, if pesticide use predominantly underlies observed declines, we would expect stronger responses at lower elevations (near farms), yet we observed a stronger decline at higher elevations (see S1 Fig), against the flow direction where agricultural run-offs are unlikely.

Climate change threatens tropical montane amphibians [47] and cannot be ruled out as a potential factor driving observed changes to the anuran assemblages. While climate data are limited for our study sites, our *ad hoc* field observations suggest that climate conditions were fairly consistent (e.g. length and strength of the dry season, precipitation etc.) over this study period. The observed changes could also be due to natural population fluctuations [48]. However, as the decline was not limited to a single taxon or to species with similar environmental requirements and life-histories (e.g. tree frogs vs. leaf litter frogs; direct-developing vs. larval development) we consider natural population fluctuations as an improbable explanation.

Because *Bd* prefers cooler and moist areas [49,50], species associated with streams at higher elevations have an increased susceptibility [51–53], causing stronger *Bd*-induced declines at higher elevations [44,54,55]. In our study, the negative effect of pond availability on sampling success might be simply due to an increase of study sites with ponds visited over time (percentage of visited sites with pond per year: t = -2.86, df = 7, p < 0.05, n = 9). Although there was no significant interaction of elevation and study year in the model (see Table 1), separate analysis revealed a stronger decline over time at higher elevations (see S1 Fig). Given this phenomenon and the other available data, *Bd* seems the most plausible driver of the change in anuran communities observed on these mountains during the past decade, possibly being reinforced by other stressors like habitat degradation, pollution or change in microclimatic conditions. Individual species respond differently to *Bd* (e.g. [56,57], this study). This may be the result of differences in microhabitats and life-history traits [58–60], behaviour [61–64], condition and life-stage [65], skin peptides [66], and even associated microbiota [67]. Species of the genus *Phrynobatrachus* seem particularly susceptible as they were rarely or never observed after the emergence of *Bd* on both mountains. In our study areas, these species inhabit the leaf litter and breed in temporary waters and medium-sized streams where their tadpoles develop [68]. Their preferred moist habitats and close association with streams correspond well to the environmental requirements of *Bd* [49,50] and might increase their susceptibility to infection. While stream-dwelling anuran species are particularly affected by *Bd* [60,69], not all such species at our study sites declined, including species of *Leptopelis*, *Leptodactylodon*, and *Astylosternus*. Here, the use of different microhabitats might result in different exposure to *Bd*. *Astylosternus* tadpoles, for example, live in fast-flowing and sandy streams ([70], personal observation), whereas *Phrynobatrachus* tadpoles occur in slow-moving parts of streams and adjacent pools, hiding between stones and in the leaf litter [68]. Many *Leptopelis* species included here are largely arboreal, but deposit eggs in humid soil [71] and thus may have less exposure as adults to *Bd* than species found in streams and ponds. In contrast, *Leptodactylodon* are often found close to or even in the streams where they reproduce. They hide in muddy burrows and piles of dead leaves and branches along the streams [(personal observation; [72]). Their tadpoles dwell mostly beneath stones and in the leaf litter of slower-moving streams [73]. The microhabitats of both life-cycle stages thus meet the environmental requirements of *Bd*. Their resistance might be due to reasons like skin structure, peptide composition, behaviour and microbiota, but this remains unknown. In summary, when considering the different possibilities for the decline with the evidence (species-specific reactions, habitat requirements, elevational distribution), *Bd* still seems to be the most likely factor.

It is unknown whether the emergence of *Bd* in the highlands of Cameroon is novel or endemic [74]. The first studies of recently collected material from Cameroon failed to detect *Bd* [25,26,75]. However, the data presented here support a long-term presence of *Bd* in the lowlands [28] though at a much lower prevalence than recently documented from the earliest 20^th^ century in the Atlantic coastal forests of Brazil [76]. We do not know the *Bd*-strain of the historical positives, although sequences of recent *Bd* positives suggest a relationship to the hypervirulent global panzootic lineage ([77], DCB & SG unpublished data). In the case of endemic emergence, *Bd* might not have been detected by past studies [25,26] because of low-intensity infections leading to false negative results [31] or strains and other chytrid pathogens which are not detectable with protocols currently applied [4]. Alternatively, the few early reports of *Bd* in Cameroon could be the result of false positives. Infection intensities observed in this study were not as high as those observed for species in *Bd*-induced declines elsewhere [78]. This may be affected by our surveys occurring during periods of low virulence prior to or following disease outbreak, or pathogenic DNA becoming depleted during transport back to the laboratory in the tropical climate [79]. The low sample sizes and differential diagnostic sampling regime (museum vs. toe-clip vs. swab) of historic samples also makes robust inference of historical absence of *Bd* in the mountains difficult. To unambiguously confirm the association of *Bd* with the observed declines, further examination of museum specimens, infection experiments, and other factors that might change host-parasite dynamics are required.

Because tropical mountains host large numbers of endemic species, die-offs on single mountains may lead to the extinction of entire species. This may include the two Cameroonian mountains studied here and other mountains in the Cameroon Volcanic Line. Urgent conservation strategies are needed for conserving the frog species endemic to these mountains. Captive breeding programs are conceivable for critically endangered species to facilitate later reintroductions or translocations following disease-induced mortality events [80]. Another approach could be antifungal treatments of species in risk [81] possibly combined with environmental disinfection which successfully eliminated *Bd* infection in pond breeding amphibian species [82]. However, as susceptibility to lethal diseases might be strongly linked to the stress experienced by a species, the protection of the remaining habitats is also critical to conserving the rich amphibian assemblages on both mountains.

## Supporting Information

S1 Fig Sampling success in response to study year and elevation and its interaction. Sampling success (species detected per man hour) decreased on both mountains significantly with study year (Spearman Rank Correlation: Manengouba (grey): rho = -0.48, p < 0.0001; Oku (red): rho = -0.31, p < 0.01), elevation (Manengouba: rho = -0.12, p < 0.05; Oku: rho = -0.33, p < 0.01), and the interaction of elevation and study year (Manengouba: rho = -0.49, p < 0.0001; Oku: rho = -0.39, p < 0.001); elevation and year are scaled form 0 to 1, respectively.
(DOCX)

S2 Fig Frequency of both genera and species in the study regions over time. Proportion of search events that a genus or species of frog was present per year on Mt. Manengouba (A–C) and Mt. Oku (D). Bars before the detection of *Bd* are colored white, and after *Bd* black; number of search events Mt. Manengouba: 2004 = 5, 2005 = 25, 2006 = 66, 2007 = 31, 2008 = 70, 2010 = 71, 2011 = 63, 2012 = 35, before Bd = 366, after Bd = 98; Mt. Oku: 2004 = 2, 2006 = 97, 2008 = 18, 2009 = 21, 2010 = 3, 2012 = 52, before *Bd* = 99, after *Bd* = 94. For each plot, the rightmost two bars show the proportion of search events that a genus or species was detected before and after the first *Bd* record on that mountain. The genera and species shown here are restricted to those recorded during at least 10 search events. See Fig 3 for additional genera and species from Mt. Manengouba.
(DOCX)

S1 Table Voucher specimens. Individuals collected at Mt. Manengouba, Mt. Oku and neighboring localities for the purpose of other study questions. Those vouchers were tested for the study presented herein to obtain a continuous Chronology of *Bd* records.
(DOCX)

S2 Table Chronology of *Bd* records and infection load. Field-collected and museum samples from Cameroon tested for *Bd*. Given is an identification number (Museums Catalogue or Field Number), species identity, year of collection, screening results (positive or negative, zoospore equivalent, ZE and genomic equivalent, GE), and collection site. Individuals tested positive are highlighted in yellow.
(XLSX)

S3 Table Summary of the chronology of *Bd* emergence in Cameroon based on screening field-collected and museum samples. Field-collected and museum samples from Cameroon tested for *Bd* summarized by collection site and year. Confidence intervals for prevalence are calculated using R package ‘PropCIs’.
(DOCX)
