# Supplementary material for: Dramatic Declines of Montane Frogs in a Central African Biodiversity Hotspot
Source: PLoS One. 2016 May 5;11(5):e0155129. doi: 10.1371/journal.pone.0155129 (PMC4858272; doi:10.1371/journal.pone.0155129)

**S1 Fig**

**Sampling success in response to study year and elevation and its interaction.** Sampling success (species detected per man hour) decreased on both mountains significantly with study year (Spearman Rank Correlation: Manengouba (grey): rho = -0.48, p < 0.0001; Oku (red): rho = -0.31, p < 0.01), elevation (Manengouba: rho = -0.12, p < 0.05; Oku: rho = -0.33, p < 0.01), and the interaction of elevation and study year (Manengouba: rho = -0.49, p < 0.0001; Oku: rho = -0.39, p < 0.001); elevation and year are scaled form 0 to 1, respectively.


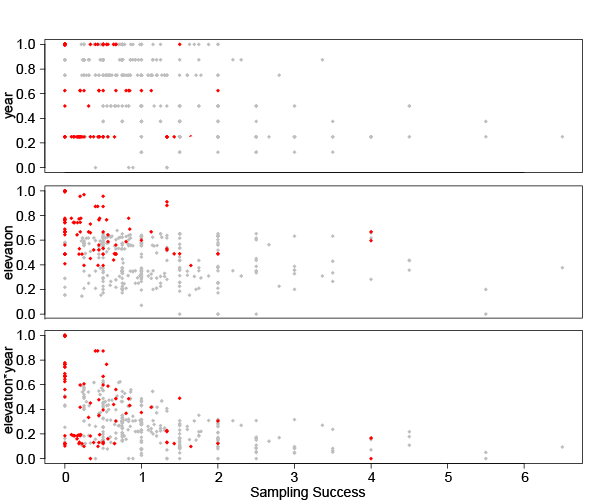

Supplement: S1 Fig — Sampling success (species detected per man hour) decreased on both mountains significantly with study year (Spearman Rank Correlation: Manengouba (grey): rho = -0.48, p < 0.0001; Oku (red): rho = -0.31, p < 0.01), elevation (Manengouba: rho = -0.12, p < 0.05; Oku: rho = -0.33, p < 0.01), and the interaction of elevation and study year (Manengouba: rho = -0.49, p < 0.0001; Oku: rho = -0.39, p < 0.001); elevation and year are scaled form 0 to 1, respectively. (DOCX) [file pone.0155129.s001.docx]
