# Supplementary material for: Dramatic Declines of Montane Frogs in a Central African Biodiversity Hotspot
Source: PLoS One. 2016 May 5;11(5):e0155129. doi: 10.1371/journal.pone.0155129 (PMC4858272; doi:10.1371/journal.pone.0155129)

**S2 Fig**

**Frequency of both genera and species in the study regions over time.** Proportion of search events that a genus or species of frog was present per year on Mt. Manengouba (A–C) and Mt. Oku (D). Bars before the detection of Bd are colored white, and after Bd black; number of search events Mt. Manengouba: 2004 = 5, 2005 = 25, 2006 = 66, 2007 = 31, 2008 = 70, 2010 = 71, 2011 = 63, 2012 = 35, before Bd = 366, after Bd = 98; Mt. Oku: 2004 = 2, 2006 = 97, 2008 = 18, 2009 = 21, 2010 = 3, 2012 = 52, before Bd = 99, after Bd = 94. For each plot, the rightmost two bars show the proportion of search events that a genus or species was detected before and after the first Bd record on that mountain. The genera and species shown here are restricted to those recorded during at least 10 search events. See Fig. 3 for additional genera and species from Mt. Manengouba.


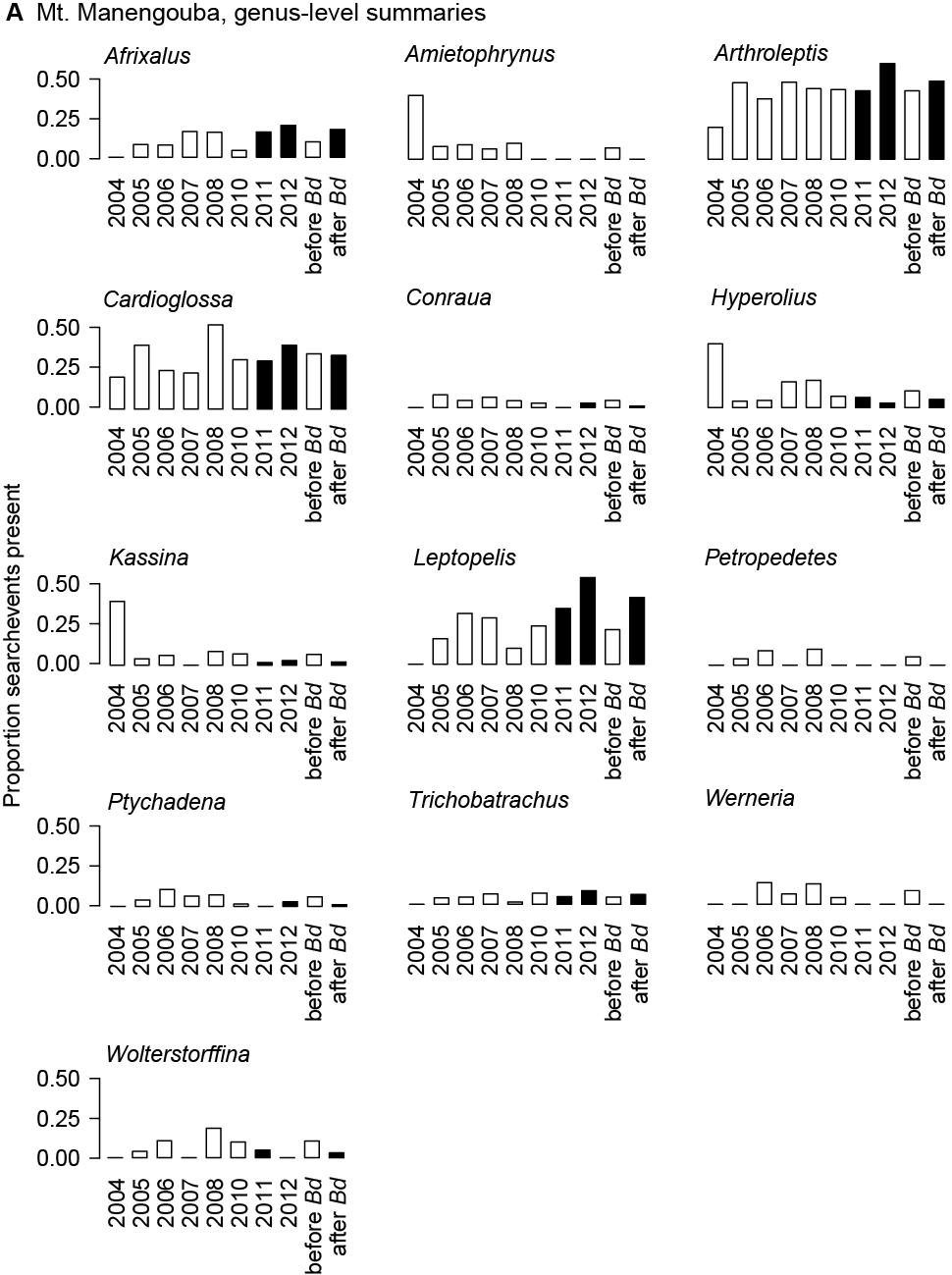


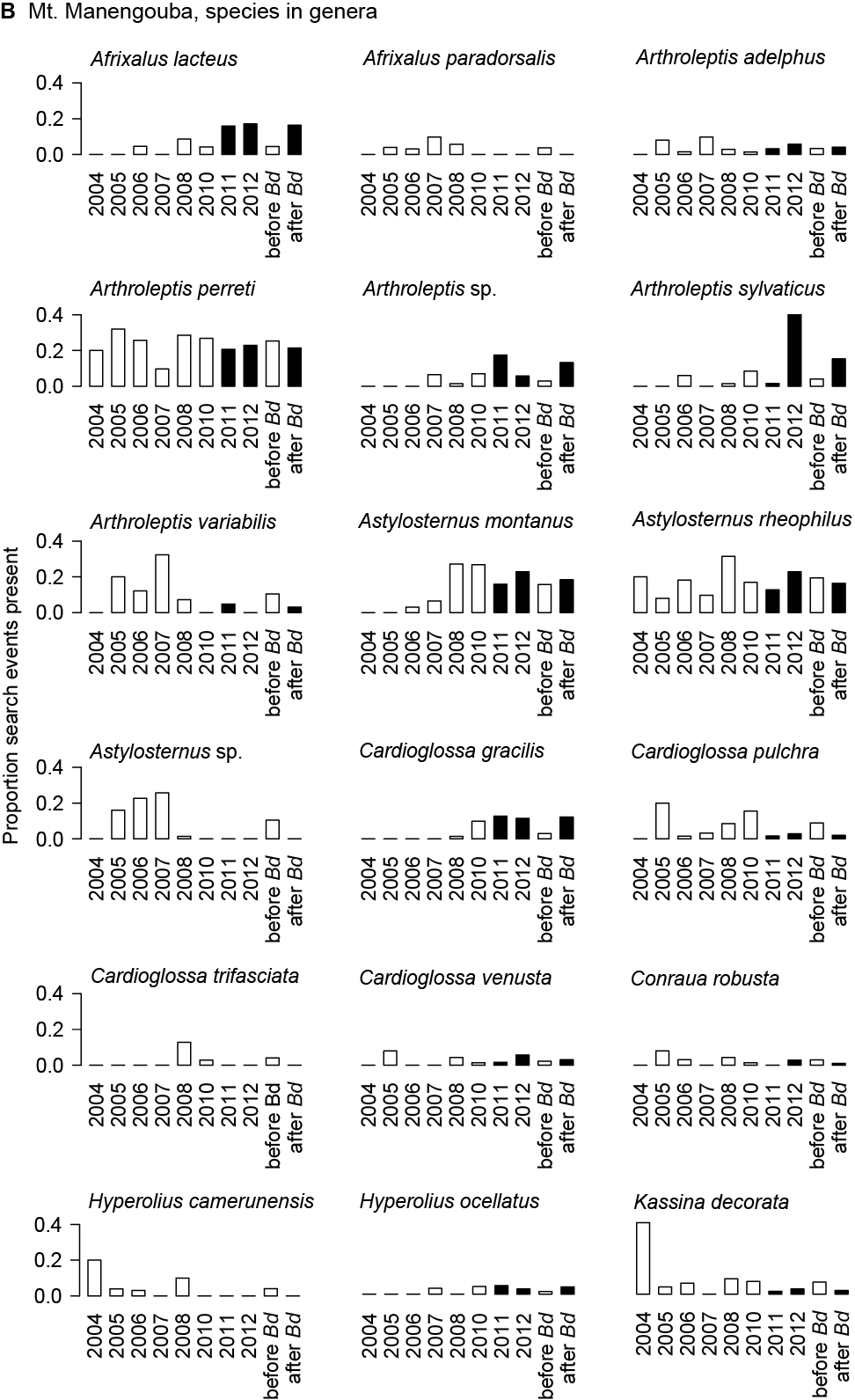


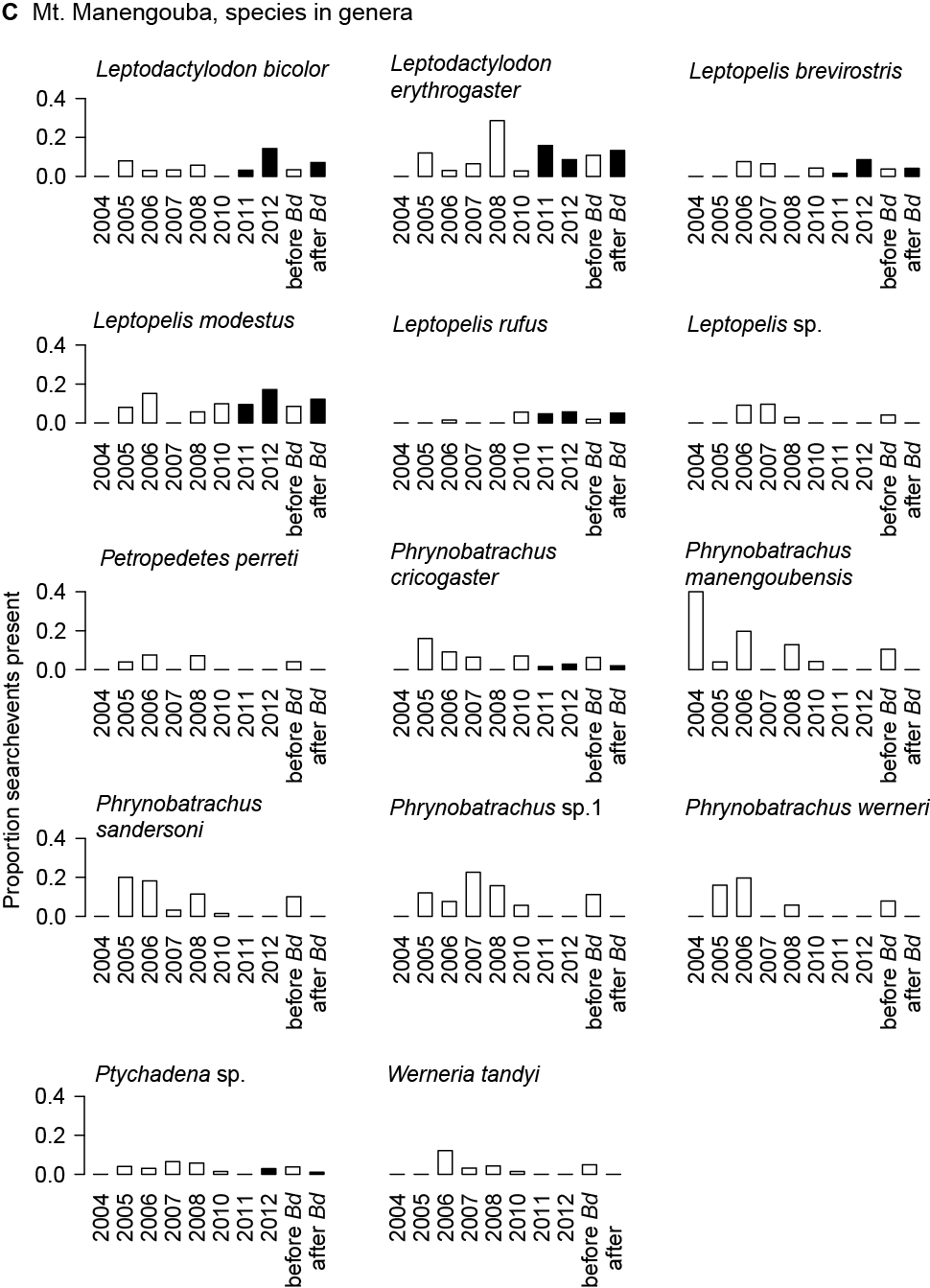


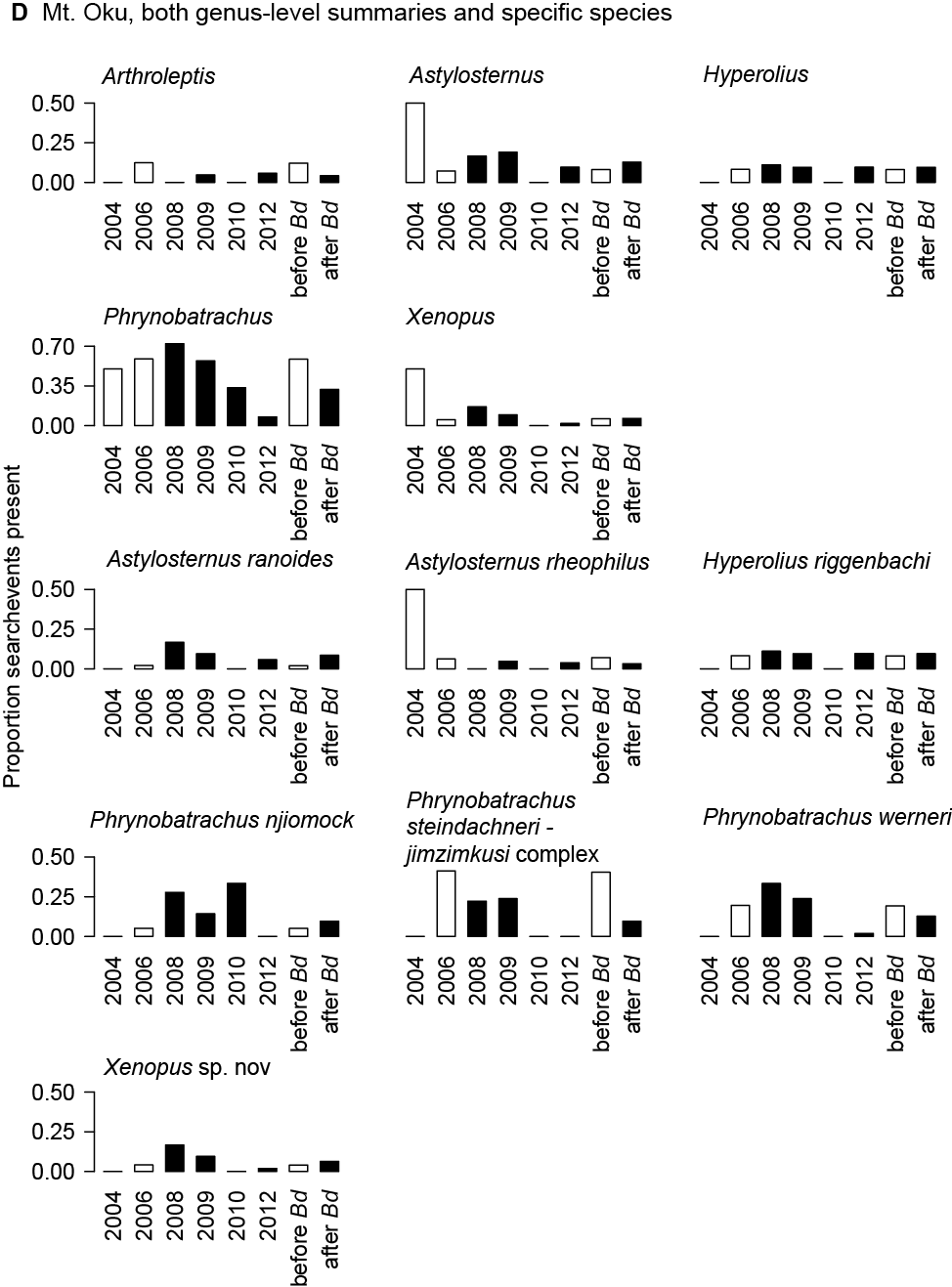

Supplement: S2 Fig — Proportion of search events that a genus or species of frog was present per year on Mt. Manengouba (A–C) and Mt. Oku (D). Bars before the detection of Bd are colored white, and after Bd black; number of search events Mt. Manengouba: 2004 = 5, 2005 = 25, 2006 = 66, 2007 = 31, 2008 = 70, 2010 = 71, 2011 = 63, 2012 = 35, before Bd = 366, after Bd = 98; Mt. Oku: 2004 = 2, 2006 = 97, 2008 = 18, 2009 = 21, 2010 = 3, 2012 = 52, before Bd = 99, after Bd = 94. For each plot, the rightmost two bars show the proportion of search events that a genus or species was detected before and after the first Bd record on that mountain. The genera and species shown here are restricted to those recorded during at least 10 search events. See Fig 3 for additional genera and species from Mt. Manengouba. (DOCX) [file pone.0155129.s002.docx]
