# Supplementary material for: Dramatic Declines of Montane Frogs in a Central African Biodiversity Hotspot
Source: PLoS One. 2016 May 5;11(5):e0155129. doi: 10.1371/journal.pone.0155129 (PMC4858272; doi:10.1371/journal.pone.0155129)
Supplement: S1 Table — Individuals collected at Mt. Manengouba, Mt. Oku and neighboring localities for the purpose of other study questions. Those vouchers were tested for the study presented herein to obtain a continuous Chronology of Bd records. (DOCX) [file pone.0155129.s003.docx]

**Table S1**

**Voucher specimens.** Individuals collected at Mt. Manengouba, Mt. Oku and neighboring localities for the purpose of other study questions. Those vouchers were tested for the study presented herein to obtain a continuous Chronology of *Bd* records.

| **Family** | **Genus** | **Species** | **# Vouchers** |
| --- | --- | --- | --- |
| Arthroleptidae | *Arthroleptis* | *palava* | 1 |
| Arthroleptidae | *Arthroleptis* | *poecilonotus* | 2 |
| Arthroleptidae | *Arthroleptis* | *variabilis* | 15 |
| Arthroleptidae | *Astylosternus* | *perreti* | 3 |
| Arthroleptidae | *Astylosternus* | *rheophilus* | 13 |
| Arthroleptidae | *Astylosternus* | sp*.* | 4 |
| Arthroleptidae | *Cardioglossa* | *manengouba* | 4 |
| Arthroleptidae | *Cardioglossa* | *melanogaster* | 2 |
| Arthroleptidae | *Cardioglossa* | *oreas* | 2 |
| Arthroleptidae | *Cardioglossa* | *pulchra* | 2 |
| Arthroleptidae | *Cardioglossa* | *schioetzi* | 6 |
| Arthroleptidae | *Leptodactylodon* | *mertensi* | 7 |
| Arthroleptidae | *Leptodactylodon* | *perreti* | 7 |
| Arthroleptidae | *Leptopelis* | sp*.* | 1 |
| Bufonidae | *Amietophrynus* | *maculatus* | 7 |
| Bufonidae | *Amietophrynus* | *villiersi* | 1 |
| Bufonidae | *Werneria* | *tandyi* | 2 |
| Hyperoliidae | *Hyperolius* | *balfouri viridistriatus* | 5 |
| Hyperoliidae | *Hyperolius* | *guttulatus* | 1 |
| Hyperoliidae | *Kassina* | *decorata* | 8 |
| Petropedetidae | *Petropedetes* | *perreti* | 1 |
| Phrynobatrachidae | *Phrynobatrachus* | *chukuchuku* | 11 |
| Phrynobatrachidae | *Phrynobatrachus* | *cricogaster* | 1 |
| Phrynobatrachidae | *Phrynobatrachus* | *manengoubensis* | 1 |
| Phrynobatrachidae | *Phrynobatrachus* | sp*.* | 1 |
| Phrynobatrachidae | *Phrynobatrachus* | *steindachneri -* complex | 51 |
| Pipidae | *Xenopus* | *longipes* | 26 |
