# Supplementary material for: Dramatic Declines of Montane Frogs in a Central African Biodiversity Hotspot
Source: PLoS One. 2016 May 5;11(5):e0155129. doi: 10.1371/journal.pone.0155129 (PMC4858272; doi:10.1371/journal.pone.0155129)
Supplement: S3 Table — Field-collected and museum samples from Cameroon tested for Bd summarized by collection site and year. Confidence intervals for prevalence are calculated using R package ‘PropCIs’. (DOCX) [file pone.0155129.s005.docx]

**S3 Table**

**Summary of the chronology of *Bd* emergence in Cameroon based on screening field-collected and museum samples.** Field-collected and museum samples from Cameroon tested for *Bd* summarized by collection site and year. Confidence intervals for prevalence are calculated using R package ‘PropCIs, study region: M: Mt. Manengouba, O: Mt. Oku

| **Year** | **Locality** | **Study Region** | **Sampling technique** | **# sampled** | **# positive** | **Prevalence** | **Lower 95% CI** | **Upper 95% CI** | **Source** |
| --- | --- | --- | --- | --- | --- | --- | --- | --- | --- |
| **Adamaua Region** | |  |  |  |  |  |  |  |  |
| 2006 | Sambolabbo |  | Museum | 3 | 0 | 0.00 | 0.000 | 0.707 | This study |
|  |  |  |  |  |  |  |  |  |  |
| **Centre Region** | |  |  |  |  |  |  |  |  |
| 1972 | Mt Kala |  | Museum | 3 | 0 | 0.00 | 0.000 | 0.707 | This study |
| 1973 | Mt Kala |  | Museum | 1 | 0 | 0.00 | 0.000 | 0.975 | This study |
| 2008 | Ndikiniméki |  | Swab | 74 | 43 | 0.58 | 0.461 | 0.695 | Doherty-Bone et al. 2013 |
| 2011 | Meganme |  | Swab | 16 | 5 | 0.31 | 0.110 | 0.587 | This study |
| 2011 | Yaoundé |  | Swab | 30 | 8 | 0.27 | 0.123 | 0.459 | This study |
| 2011 | pond on trail near Meganme |  | Swab | 27 | 3 | 0.11 | 0.023 | 0.291 | This study |
| 2011 | Sanaga River, fisherman's camp |  | Swab | 3 | 1 | 0.33 | 0.008 | 0.906 | This study |
|  |  |  |  |  |  |  |  |  |  |
| **East Region** | |  |  |  |  |  |  |  |  |
| 1930 | Bitye, Dja River |  | Museum | 4 | 0 | 0.00 | 0.000 | 0.602 | This study |
| 1934 | Batouri |  | Museum | 8 | 1 | 0.13 | 0.003 | 0.527 | Soto-Azat et al. 2010 |
| 1994 | Dja Reserve, Boumir Camp |  | Museum | 149 | 0 | 0.00 | 0.000 | 0.024 | This study |
| 2009 | Doumo-Pierre |  | Swab | 112 | 0 | 0.00 | 0.000 | 0.032 | Doherty-Bone et al. 2013 |
| 2013 | Mebam |  | Swab | 30 | 3 | 0.10 | 0.021 | 0.265 | This study |
| 2013 | Zoulabot II |  | Swab | 25 | 24 | 0.96 | 0.796 | 0.999 | This study |
| 2013 | Ngoyla |  | Swab | 23 | 3 | 0.13 | 0.028 | 0.336 | This study |
| 2013 | Doumzok |  | Swab | 22 | 3 | 0.14 | 0.029 | 0.349 | This study |
|  |  |  |  |  |  |  |  |  |  |
| **Littoral Region** | |  |  |  |  |  |  |  |  |
| 1959 | Manengouba, Nsoung | M | Museum | 2 | 0 | 0.00 | 0.000 | 0.841 | This study |
| 1960 | Manengouba, Nsoung | M | Museum | 2 | 0 | 0.00 | 0.000 | 0.841 | This study |
| 1972 | Bakaka Forest Reserve |  | Museum | 1 | 0 | 0.00 | 0.000 | 0.975 | This study |
| 2004 | Mt Manengouba | M | Museum | 22 | 0 | 0.00 | 0.000 | 0.154 | This study |
| **Year** | **Locality** | **Study Region** | **Sampling technique** | **# sampled** | **# positive** | **Prevalence** | **Lower 95% CI** | **Upper 95% CI** | **Source** |
| 2006 | Mt Mbam | M | Museum | 25 | 0 | 0.00 | 0.000 | 0.137 | This study |
| 2006 | Mt Manengouba | M | Museum | 13 | 0 | 0.00 | 0.000 | 0.247 | This study |
| 2006 | Manengouba, Nsoung | M | Museum | 25 | 0 | 0.00 | 0.000 | 0.137 | This study |
| 2007 | Mt Manengouba | M | Swab | 248 | 0 | 0.00 | 0.000 | 0.014 | Doherty-Bone et al .2013 |
| 2008 | Mt. Manengouba | M | Swab | 43 | 0 | 0.00 | 0.000 | 0.082 | Doherty-Bone et al .2013 |
| 2011 | Manengouba village | M | Swab | 18 | 4 | 0.22 | 0.064 | 0.476 | Doherty-Bone et al .2013 |
| 2011 | Mt. Manengouba | M | Swab | 42 | 13 | 0.31 | 0.176 | 0.471 | Doherty-Bone et al .2013 |
| 2011 | Ebonemin, Mt Manengouba | M | Swab | 35 | 4 | 0.11 | 0.032 | 0.267 | Doherty-Bone et al .2013 |
| 2013 | Manjo | M | Swab | 110 | 35 | 0.32 | 0.233 | 0.414 | This study |
| 2013 | Mt. Manengouba | M | Swab | 33 | 27 | 0.82 | 0.645 | 0.930 | This study |
|  |  |  |  |  |  |  |  |  |  |
| **North West Region** | |  |  |  |  |  |  |  |  |
| 1930 | Bamenda | O | Museum | 2 | 0 | 0.00 | 0.000 | 0.841 | This study |
| 1952 | Bamenda | O | Museum | 4 | 0 | 0.00 | 0.000 | 0.602 | This study |
| 1970 | Bamenda | O | Museum | 9 | 0 | 0.00 | 0.000 | 0.336 | This study |
| 1984 | Lake Oku | O | Museum | 44 | 0 | 0.00 | 0.000 | 0.080 | Soto-Azat et al. 2010 |
| 2004 | Lake Oku | O | Museum | 28 | 0 | 0.00 | 0.000 | 0.123 | This study |
| 2006 | Abuh | O | Toe clip | 11 | 0 | 0.00 | 0.000 | 0.284 | Doherty-Bone et al .2008 |
| 2006 | Anyafouma | O | Toe clip | 97 | 0 | 0.00 | 0.000 | 0.037 | Doherty-Bone et al .2008 |
| 2006 | Anyajua | O | Toe clip | 1 | 0 | 0.00 | 0.000 | 0.975 | Doherty-Bone et al. 2008 |
| 2006 | Elemighong | O | Toe clip | 68 | 0 | 0.00 | 0.000 | 0.053 | Doherty-Bone et al .2008 |
| 2006 | Lake Oku | O | Toe clip | 79 | 0 | 0.00 | 0.000 | 0.046 | Doherty-Bone et al .2008 |
| 2006 | Fundong | O | Toe clip | 1 | 0 | 0.00 | 0.000 | 0.975 | Doherty-Bone et al .2008 |
| 2006 | Elak Oku | O | Museum | 20 | 0 | 0.00 | 0.000 | 0.168 | This study |
| 2006 | Lake Awing | O | Museum | 6 | 0 | 0.00 | 0.000 | 0.460 | This study |
| 2006 | Lake Oku | O | Museum | 13 | 0 | 0.00 | 0.000 | 0.247 | This study |
| 2006 | Emfe-mhi Forest, Mt Oku | O | Museum | 6 | 0 | 0.00 | 0.000 | 0.460 | This study |
| 2006 | Mt Oku summit | O | Museum | 12 | 0 | 0.00 | 0.000 | 0.265 | This study |
| 2006 | Obang | O | Museum | 2 | 0 | 0.00 | 0.000 | 0.841 | This study |
| 2006 | Wum | O | Museum | 4 | 0 | 0.00 | 0.000 | 0.602 | This study |
| 2006 | Abuh | O | Toe clip | 11 | 0 | 0.00 | 0.000 | 0.284 | This study |
| 2006 | Anyafouma | O | Toe clip | 97 | 0 | 0.00 | 0.000 | 0.037 | This study |
| **Year** | **Locality** | **Study Region** | **Sampling technique** | **# sampled** | **# positive** | **Prevalence** | **Lower 95% CI** | **Upper 95% CI** | **Source** |
| 2006 | Anyajua | O | Toe clip | 1 | 0 | 0.00 | 0.000 | 0.975 | This study |
| 2006 | Elemighong | O | Toe clip | 68 | 0 | 0.00 | 0.000 | 0.052 | This study |
| 2006 | Lake Oku | O | Toe clip | 79 | 0 | 0.00 | 0.000 | 0.046 | This study |
| 2006 | Fundong | O | Toe clip | 1 | 0 | 0.00 | 0.000 | 0.975 | This study |
| 2008 | Elak Oku | O | Swab | 28 | 8 | 0.29 | 0.132 | 0.487 | Doherty-Bone et al .2013 |
| 2008 | Elemighong | O | Swab | 11 | 8 | 0.73 | 0.390 | 0.940 | Doherty-Bone et al .2013 |
| 2008 | Jikijem | O | Swab | 1 | 0 | 0.00 | 0.000 | 0.975 | Doherty-Bone et al .2013 |
| 2008 | Kedzem-Mawes Forest | O | Swab | 1 | 0 | 0.00 | 0.000 | 0.975 | Doherty-Bone et al .2013 |
| 2008 | Kissotin | O | Swab | 13 | 7 | 0.54 | 0.251 | 0.808 | Doherty-Bone et al .2013 |
| 2008 | Lake Oku | O | Swab | 93 | 5 | 0.05 | 0.018 | 0.121 | Doherty-Bone et al .2013 |
| 2008 | Mbam-Oku | O | Swab | 1 | 0 | 0.00 | 0.000 | 0.975 | Doherty-Bone et al .2013 |
| 2008 | Elak Oku | O | Swab | 28 | 8 | 0.29 | 0.132 | 0.487 | This study |
| 2008 | Elemighong | O | Swab | 11 | 8 | 0.73 | 0.390 | 0.940 | This study |
| 2008 | Jikijem | O | Swab | 1 | 0 | 0.00 | 0.000 | 0.975 | This study |
| 2008 | Kedzem-Mawes Forest | O | Swab | 1 | 0 | 0.00 | 0.000 | 0.975 | This study |
| 2008 | Kissotin | O | Swab | 13 | 7 | 0.54 | 0.251 | 0.807 | This study |
| 2008 | Lake Oku | O | Swab | 93 | 5 | 0.05 | 0.018 | 0.121 | This study |
| 2008 | Mbam-Oku | O | Swab | 1 | 0 | 0.00 | 0.000 | 0.975 | This study |
| 2009 | Afua Swamp | O | Swab | 2 | 0 | 0.00 | 0.000 | 0.841 | Doherty-Bone et al. 2013 |
| 2009 | Lake Oku | O | Swab | 11 | 0 | 0.00 | 0.000 | 0.284 | Doherty-Bone et al .2013 |
| 2009 | Anyafouma | O | Swab | 1 | 0 | 0.00 | 0.000 | 0.975 | Doherty-Bone et al .2013 |
| 2009 | Kissotin | O | Swab | 1 | 0 | 0.00 | 0.000 | 0.975 | Doherty-Bone et al .2013 |
| 2009 | Oku summit | O | Swab | 1 | 0 | 0.00 | 0.000 | 0.975 | Doherty-Bone et al .2013 |
| 2009 | Afua Swamp | O | Swab | 2 | 0 | 0.00 | 0.000 | 0.841 | This study |
| 2009 | Lake Oku | O | Swab | 11 | 0 | 0.00 | 0.000 | 0.284 | This study |
| 2009 | Anyafouma | O | Swab | 1 | 0 | 0.00 | 0.000 | 0.975 | This study |
| 2009 | Kissotin | O | Swab | 1 | 0 | 0.00 | 0.000 | 0.975 | This study |
| 2009 | Oku summit | O | Swab | 1 | 0 | 0.00 | 0.000 | 0.975 | This study |
| 2010 | Mt Fungom |  | Swab | 34 | 0 | 0.00 | 0.000 | 0.103 | Balaz et al. 2013 |
| 2011 | 05 51 20.64 N, 10 09 30.72 E | O | Swab | 16 | 12 | 0.75 | 0.476 | 0.927 | This study |
| 2011 | 05 54 32.46 N, 10 09 40.56 E | O | Swab | 1 | 1 | 1.00 | 0.025 | 1.000 | This study |
| 2011 | 06 32 55.02 N, 10 45 35.94 E | O | Swab | 11 | 11 | 1.00 | 0.715 | 1.000 | This study |
| **Year** | **Locality** | **Study Region** | **Sampling technique** | **# sampled** | **# positive** | **Prevalence** | **Lower 95% CI** | **Upper 95% CI** | **Source** |
| 2011 | Bamenda | O | Swab | 30 | 15 | 0.50 | 0.313 | 0.687 | This study |
| 2011 | Nkambe | O | Swab | 12 | 10 | 0.83 | 0.516 | 0.979 | This study |
| 2011 | Lake Nyos | O | Swab | 3 | 2 | 0.67 | 0.094 | 0.991 | This study |
| 2011 | Lake Oku | O | Swab | 10 | 7 | 0.70 | 0.347 | 0.933 | This study |
| 2011 | Bafut | O | Swab | 19 | 0 | 0.00 | 0.000 | 0.176 | This study |
| 2011 | Obang | O | Swab | 3 | 1 | 0.33 | 0.008 | 0.906 | This study |
| 2013 | Bamenda | O | Swab | 12 | 11 | 0.92 | 0.615 | 0.998 | This study |
|  |  |  |  |  |  |  |  |  |  |
| **South Region** | |  |  |  |  |  |  |  |  |
| 1908 | Kribi |  | Museum | 1 | 0 | 0.00 | 0.000 | 0.975 | This study |
| 1910 | Kribi |  | Museum | 1 | 0 | 0.00 | 0.000 | 0.975 | This study |
| 1911 | Lolodorf |  | Museum | 4 | 0 | 0.00 | 0.000 | 0.602 | This study |
| 1960 | Sangmelima |  | Museum | 7 | 1 | 0.14 | 0.003 | 0.579 | This study |
| 1961 | Sangmelima |  | Museum | 6 | 0 | 0.00 | 0.000 | 0.460 | This study |
| 1966 | Lolodorf |  | Museum | 32 | 0 | 0.00 | 0.000 | 0.108 | This study |
| 1981 | Nyabessan |  | Museum | 5 | 0 | 0.00 | 0.000 | 0.522 | This study |
| 1981 | Ebolowa |  | Museum | 8 | 0 | 0.00 | 0.000 | 0.370 | This study |
| 1981 | Avundi |  | Museum | 35 | 0 | 0.00 | 0.000 | 0.100 | This study |
| 2007 | Campo National Park |  | Toe clip & museum | 130 | 0 | 0.00 | 0.000 | 0.028 | Doherty-Bone et al. 2013 |
| 2010 | Lobeke National Park |  | Swab | 70 | 1 | 0.01 | 0.000 | 0.077 | Balaz et al. 2012 |
| 2013 | Metomassala |  | Swab | 1 | 0 | 0.00 | 0.000 | 0.975 | This study |
| 2013 | Sangmelima |  | Swab | 30 | 13 | 0.43 | 0.255 | 0.626 | This study |
| 2013 | Mekas |  | Swab | 100 | 0 | 0.00 | 0.000 | 0.036 | This study |
|  |  |  |  |  |  |  |  |  |  |
| **South West Region** | |  |  |  |  |  |  |  |  |
| 1980 | Mundemba |  | Museum | 4 | 0 | 0.00 | 0.000 | 0.602 | This study |
| 2004 | Etome, Mt Cameroon |  | Museum | 1 | 0 | 0.00 | 0.000 | 0.975 | This study |
| 2004 | Ntale, Banyang Mbo | M | Museum | 9 | 0 | 0.00 | 0.000 | 0.336 | This study |
| 2004 | Ejagham Forest Reserve |  | Museum | 11 | 0 | 0.00 | 0.000 | 0.284 | This study |
| 2004 | Efolofo, Mt Cameroon |  | Museum | 3 | 0 | 0.00 | 0.000 | 0.707 | This study |
| 2007 | Mt Manengouba | M |  | 248 | 0 | 0.00 | 0.000 | 0.015 | Doherty-Bone et al. 2013 |
| **Year** | **Locality** | **Study Region** | **Sampling technique** | **# sampled** | **# positive** | **Prevalence** | **Lower 95% CI** | **Upper 95% CI** | **Source** |
| 2008 | Banga Bakundu |  | Swab | 7 | 7 | 1.00 | 0.590 | 1.000 | Doherty-Bone et al. 2013 |
| 2008 | Mt Cameroon |  | Swab | 5 | 3 | 0.60 | 0.147 | 0.947 | Doherty-Bone et al. 2013 |
| 2008 | Mundame |  | Swab | 21 | 12 | 0.57 | 0.340 | 0.782 | Doherty-Bone et al. 2013 |
| 2009 | Mt Kupe |  | Toe clip | 82 | 0 | 0.00 | 0.000 | 0.044 | Doherty-Bone et al. 2013 |
| 2009 | Rumpi Hills |  | Toe clip & museum | 144 | 0 | 0.00 | 0.000 | 0.025 | Doherty-Bone et al. 2013 |
| 2011 | Nkack, Mt Manengouba | M | Swab | 10 | 2 | 0.20 | 0.025 | 0.556 | This study |
| 2011 | Nkikoh | M | Swab | 3 | 0 | 0.00 | 0.000 | 0.707 | This study |
| 2011 | Pola | M | Swab | 1 | 0 | 0.00 | 0.000 | 0.975 | This study |
| 2013 | Ekenzu, Large stream | M | Swab | 13 | 6 | 0.46 | 0.192 | 0.749 | This study |
| 2013 | Edib | M | Swab | 33 | 21 | 0.64 | 0.451 | 0.796 | This study |
| 2013 | Small Koto |  | Swab | 55 | 26 | 0.53 | 0.337 | 0.612 | This study |
|  |  |  |  |  |  |  |  |  |  |
| **West Region** | |  |  |  |  |  |  |  |  |
| 2004 | Mt Bamboutous |  | Museum | 20 | 0 | 0.00 | 0.000 | 0.168 | This study |
| 2006 | Mt Bamboutous |  | Museum | 26 | 0 | 0.00 | 0.000 | 0.132 | This study |
| 2008 | Dschang |  | Swab | 5 | 4 | 0.80 | 0.284 | 0.995 | Doherty-Bone et al. 2013 |
| 2011 | 05 06 31.98 N, 10 36 57.12 E |  | Swab | 3 | 1 | 0.33 | 0.008 | 0.906 | This study |
| 2011 | Balaoungao |  | Swab | 9 | 0 | 0.00 | 0.000 | 0.336 | This study |
| 2011 | Lake Nfou |  | Swab | 4 | 1 | 0.25 | 0.006 | 0.806 | This study |
| 2011 | Ngindom Coc |  | Swab | 3 | 0 | 0.00 | 0.000 | 0.707 | This study |
| 2013 | Bangoua |  | Swab | 9 | 5 | 0.56 | 0.212 | 0.863 | This study |
| 2013 | Bamboutos (1) |  | Swab | 1 | 1 | 1.00 | 0.025 | 1.000 | This study |
| 2013 | Bamboutos (2) |  | Swab | 1 | 1 | 1.00 | 0.025 | 1.000 | This study |
